# Nigerian parents and caregivers knowledge, attitude and willingness to vaccinate their children against COVID-19

**DOI:** 10.3389/fpubh.2023.1047285

**Published:** 2023-10-02

**Authors:** Azeezat Ajose, Cassandra Akinde, Azizat Ilo, Tobi Durojaiye, Yusuf Shittu, Tolani Kadiri, Bisola Raheem, Mujidat Kehinde Alamutu, Olamide Ojo, Alero Ann Roberts

**Affiliations:** ^1^Research Team, The Neo Child Initiative, Lagos, Nigeria; ^2^Lagos University Teaching Hospital, Lagos, Nigeria; ^3^Team Lead, The Neo Child Initiative, Lagos, Nigeria; ^4^Senior Clinical Research Associate, XCene Research, Lagos, Nigeria; ^5^Founder, The Neo Child Initiative, Lagos, Nigeria; ^6^Department of Medicine and Surgery, College of Medicine, University of Lagos, Lagos, Nigeria; ^7^Faculty of Dental Sciences, College of Medicine, University of Lagos, Lagos, Nigeria; ^8^Department of Community Health and Primary Care, College of Medicine, University of Lagos, Lagos, Nigeria

**Keywords:** COVID-19, vaccination, children, parent, willingness, Nigeria

## Abstract

**Objectives:**

In order to achieve herd immunity against COVID-19, a significant proportion of the population will need to be vaccinated. Experts have recommended that African children be allowed to get vaccinated to protect them from emerging variants of COVID-19 infection. This study investigated Nigerian parents and caregivers’ knowledge, attitude, and willingness to vaccinate their children against COVID-19 once the vaccines are made available to them.

**Methods:**

A cross-sectional online survey of 500 parents/caregivers was conducted in Nigeria. Participants were asked to complete a questionnaire about their sociodemographic characteristics, knowledge of and attitude toward COVID-19 infection and vaccination, willingness to vaccinate their child and factors that could influence their decision to vaccinate their child. A scoring system was used to classify the level of knowledge and attitude of participants into 2 categories, namely poor, and good. We analyzed data obtained using SPSS Version 22.

**Results:**

Majority of the participants were females (63.6%). Analysis of responses revealed good knowledge and attitude in 265 (53.0%) and 266 (53.2%) respondents, respectively. Overall, less than half of the parents/caregivers (48.4%) expressed intention to vaccinate their children against COVID-19. Factors associated with willingness to vaccinate children against COVID-19 included age greater than 40 years, male gender, residing in Southern Nigeria, having good knowledge, knowing an infected person or a vaccinated person, feeling they or their child is at risk of contracting COVID-19 infection, willingness to vaccinate self against COVID-19 and good attitude. Significant predictors of willingness to vaccinate their child include age greater than 40 years [AOR: 2.56; 95% CI = (1.14–5.76)], willingness to vaccinate self [AOR: 1016.81; 95% CI = (128.51–8045.60)] and good attitude [AOR: 6.21; 95% CI = (2.83–13.64)].

**Conclusion:**

This study revealed that parental willingness to vaccinate their children against COVID-19 is low and identified factors influencing it. It is important to develop and implement health education programs iterating the risk of children getting infected with SARS-CoV-2 and its emerging variants to ensure optimal uptake of the COVID-19 vaccine in Nigerian children.

## Introduction

The novel coronavirus disease 2019 (COVID-19) infection was first reported in December, 2019 (1). Ever since, it has continued to spread across the globe with over 680 million cases and 6 million deaths reported in 231 countries (2, 3). In Nigeria, as of March 10, 2023, the country has recorded a total number of 266,598 and 3,155 cases and deaths, respectively (4). Notably, in the past year, there have been reports of the emergence of SARS-CoV-2 variants potentially more infectious and virulent than the original strain responsible for the initial outbreak (5). At the beginning of the pandemic, public health measures were recommended to prevent and reduce the transmission of COVID-19, including frequent hand-washing, use of face masks and social distancing while vaccines were in development (1, 2).

Vaccination is a cost-effective way of boosting immunity against pathogens (2). In December 2020, the SARS-CoV-2 vaccine was rolled out with the hope that it would reduce the disease burden on healthcare systems globally and put an end to the pandemic (1). The COVID-19 vaccination program in Nigeria has been implemented in both urban and rural areas, with primary healthcare centers across all 774 local government areas providing free vaccines (6). The first batch of COVID-19 vaccines (Oxford-AstraZeneca) was received from the Japanese government in March 2021, with additional doses received from various sources throughout 2021 and 2022 (7).

As Nigeria is the most populous African country, its COVID-19 vaccination coverage will have significant subregional and regional impacts (6). Nigeria’s goal was to vaccinate at least 70% of the eligible population with COVID-19 vaccines by the end of December 2022. However, as at 20th January, 2023, the country had only fully vaccinated 65,143,040 (56%) eligible individuals, while 76,957,026 (66.4%) had received at least one vaccine dose. Vaccine hesitancy due to misinformation about the vaccine’s safety and efficacy poses a significant challenge to achieving this target. Vaccination is critical toward mitigating the outbreak as it ensures herd immunity (8). To achieve herd immunity for COVID-19 infection, there is a need to expand vaccine coverage to include children (1). A number of mRNA vaccines have been approved for use in children aged 18 years and below including Comirnaty (Pfizer-BioNTech’s BNT162b2) and Spikevax (Moderna’s mRNA-1273) (5). In 2020, the COVID-19 vaccination program in Nigeria granted a waiver for children aged 16–17 years to receive the vaccine for educational purposes, and as at February 2023, a total of 2,229,295 children had received the vaccine for this purpose (9).

Children aged 14 years and younger account for over 89 million Nigerians, almost half (44%) of the total population (10). Children and adolescents of all ages are vulnerable to SARS-CoV-2 infection. They may be at increased risk of getting infected by SARS-CoV-2 variants such as Delta and Omicron, especially if they are not vaccinated (5). COVID-19 presents with a milder course of acute illness in children compared to adults, however, there is a potential risk for severe complications that can affect their health on the long term (5). Asides these, children in LMICs like Nigeria have a higher prevalence of co-morbidities and as such, these children at increased risk of severe disease should be vaccinated against COVID-19 (11). Thus, experts have recommended that African children be allowed to get vaccinated against COVID-19 especially those with significant risk of severe disease and death, given the emerging variants of SARS-CoV-2 infection (8).

**Figure 1:**
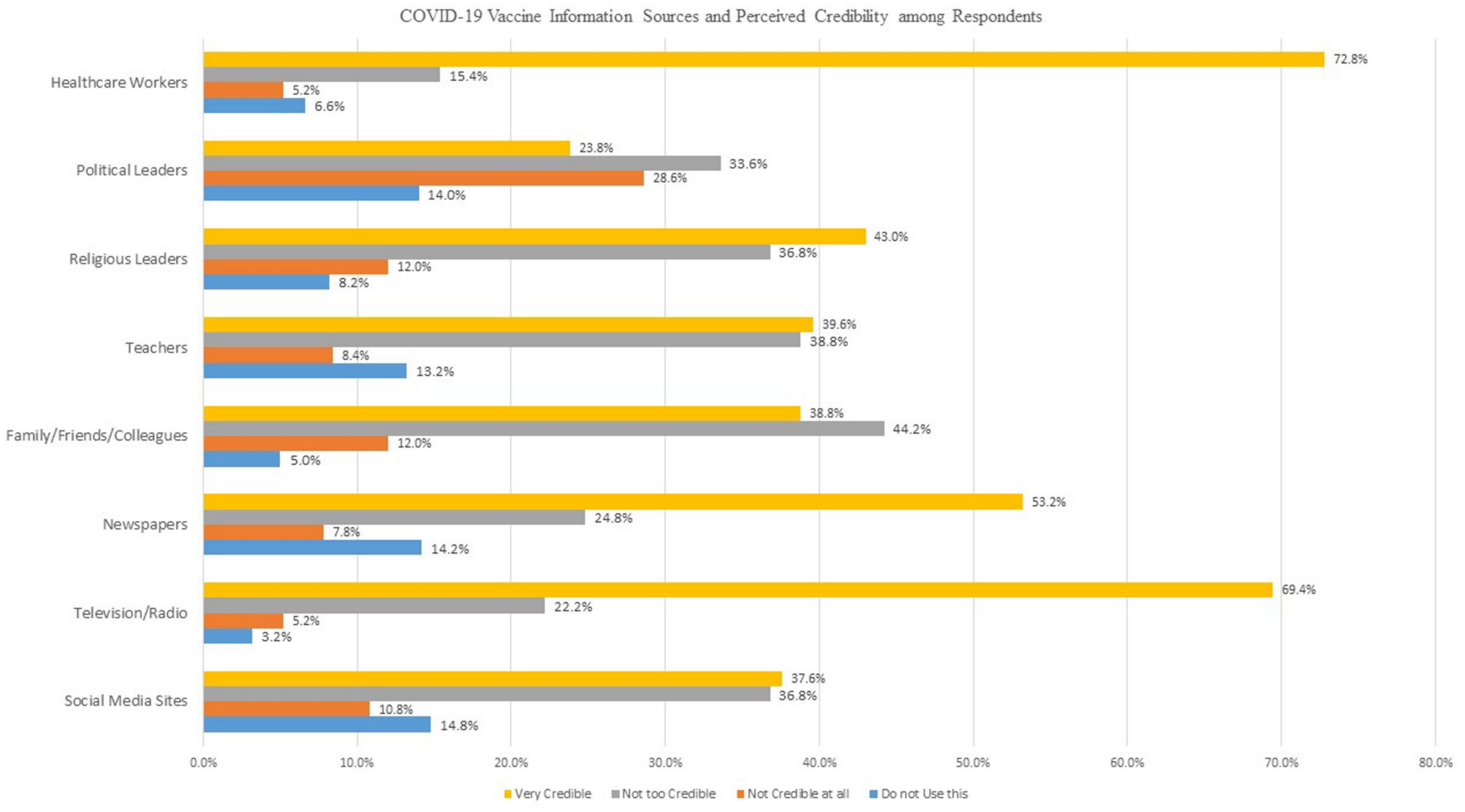
Perception of credibility of information sources regarding COVID-19.

Parents significantly influence their child’s vaccine uptake, and as such to increase the vaccination rate amongst children, parental hesitancy must be considered (1). Several research has been done to investigate parental willingness to vaccinate children since the vaccine was introduced. In a meta-analysis of 44 studies including 317,055 parents, parental intention to accept COVID-19 vaccine for their child ranged from 25.6 to 92.2%. However, the vast majority of the papers included were done in high-income countries, and it may be difficult to generalize their findings to Sub Saharan African population (12). In a rapid review carried out by Olu-Abiodun et al., in Nigeria in 2022, the vaccine acceptance rate among adults varied across the six geopolitical zones of the country, ranging from 20.0 to 58.2%. The non-acceptance of the vaccine was attributed to several factors such as propaganda, concerns over adverse effects, conspiracy theories, disbelief, and queries over vaccine safety (13). The factors that might be pertinent to the decision-making of parents regarding whether to vaccinate their children or not include vaccine side effects, conspiracy theories, disbelief, and fear of vaccine safety (Figure 1).

In June 2022, Nigeria reported fears of a fifth wave of COVID-19 outbreak amidst abysmally low vaccine uptake amongst adults. This poor uptake has been linked to vaccine hesitancy. A nationwide survey of 3,076 Nigerians reported a 50.7% vaccine acceptance rate (2). Reluctance to get vaccinated against COVID-19 can significantly impact outbreak control in the country. For minors aged under 18 years, parents and caregivers are the decision-makers and as such, it is important to determine their willingness to accept COVID-19 vaccination for their children and its determinants. This epidemiological landscape presents the urgent need to report determinants of parental willingness to vaccinate their children as identifying factors associated with parental acceptability will guide public health officials and other stakeholders on strategies to improve vaccination rates and achieve herd immunity. To address this gap in knowledge, the study was conducted by the Neo Child Initiative for Africa (TNCI), a non-governmental organization focused on improving the lives of Nigerian children through various initiatives that promote child health and education for sustainable development (14). The present study aimed to investigate the willingness of Nigerian parents and caregivers to vaccinate their children against COVID-19. We also compared the sociodemographic characteristics of parents and caregivers willing to vaccinate their child with those who were not willing to vaccinate their child.

## Materials and methods

### Study design

A country-wide cross-sectional survey was conducted, using a survey tool distributed via online media sites to all six geopolitical zones in Nigeria to assess parents and caregivers knowledge, attitude, and willingness to vaccinate their children against COVID-19 infection.

### Population and sample size

Convenient sampling was utilized for this research. Minimum sampling size was determined to be 363. This was calculated by assuming a prevalence level of 72.6 from a previous study done by Zhang et al., confidence level of 95%, margin of error of 5% and a non-response rate of 20% (15, 16).

The survey was designed on Google Form and shared on social media platforms, including Twitter, Instagram, WhatsApp, Facebook over a period of 1 month social media influencers and volunteers of the Neo Child Initiative residing in the six geopolitical zones of the country were recruited to ensure dissemination in every region. The inclusion criteria of the study were as follows: participants aged at least 18 years, parent and/caregiver of at least one child, and currently residing in Nigeria, ability to read and understand English and being a user of any of the following social media platforms – Facebook, Twitter, Instagram or WhatsApp. Participants whose children were not residing in Nigeria, and those who had no digital literacy were excluded from the study.

### Measures

The questionnaire used for this survey was developed with adaptations of relevant sections from previous studies that have sought to address similar objectives (17–20). It was initially piloted with 15 parents and caregivers selected from the six geopolitical zones with further refinement based on feedback gotten from these respondents and public health experts. The final questionnaire was then designed and disseminated using the application Google Form from the 1st of June 2021 to 28th of June 2021.

The questionnaire was self-administered and consisted of 47 questions divided into four sections: in the first part, socio-economic background was explored, including age, gender, educational level, occupation, marital status, and income. In the second part, knowledge of COVID-19 infection was asked. Respondents were also asked their opinion on the credibility of information sources for COVID-19. This question was collected on a 4-point Likert scale, ranging from not credible at all to very credible. In the third part, attitudes toward COVID-19 infection were asked, including perception of risk of infection for themselves and their child. In the fourth part, the respondents were asked if they had ever been diagnosed with COVID-19, or knew someone who had been diagnosed, whether they were willing to vaccinate themselves, and if they were willing to vaccinate their child, and reasons why they do not want to collect the vaccine. Knowledge and attitude variables were scored (A score of 1 was given for correct answers and a score of 0 for wrong answers). Using the mean values of the scores from all respondents, we created dichotomized measures for knowledge and attitude. Knowledge and attitude were categorized as good if the respondent had a score equal to or above the mean value and poor if score was lower than the mean value (19).

### Data analysis

We analyzed survey responses using the Statistical Package for the Social Sciences (SPSS) V. 21.0 (SPSS, Chicago, Illinois, United States). Descriptive statistics, including frequency, measures of central tendency and variations were used to report information about participants socio-economic characteristics, knowledge and attitudes toward COVID-19 vaccination, willingness to vaccinate their children, and factors influencing willingness to vaccinate.

Bivariate analysis was used to compare the characteristics of parents and caregivers who were willing to vaccinate their child with those who were not willing. Associations between categorical variables were analyzed using the Chi-Square and Fisher’s exact test. Bivariate logistic regression analysis was used to identify the predictors of parent and caregivers being willing to vaccinate their child and adjusted odds ratios (AORs) and 95% CIs were reported.

### Ethics approval

Ethical approval for this study was obtained from the Institutional Review Board at the Lagos University Teaching Hospital with the ethical approval number ADM/DCST/HREC/APP/4200. Implicit consent to be a part of this survey was considered when participants completed the online questionnaire.

## Results

A total of 500 adults filled the online form for data collection. There were no missing data. Most of the respondents were aged between 31 and 40 years (34.6%), Female (63.6%), married (79%) and from the Southern part of the country (89.4%). They were mostly parents (70.4%) and had tertiary level of education (54.8%). About one third of them knew a person that had been diagnosed with COVID-19 infection. Approximately half of them had good knowledge and attitude (53 and 53.2% respectively). Only a third of them (33.8%) perceived that they were at risk of COVID-19 infection and less than one-third of them (31.4%) felt that their child was at risk of COVID-19 infection. Almost all the parents/caregivers had previously vaccinated their child from birth (84.2%). Only one-tenth of them had previous side effects after vaccination (Table 1).

**Table 1:** Participant’s sociodemographic characteristics, overall knowledge, attitude, and willingness to vaccinate their child against COVID-19.

Variable		Frequency	Percentage
Age as at last birthday	18–20	16	3.2%
	21–30	124	24.8%
	31–40	173	34.6%
	41–50	118	23.6%
	51–60	58	11.6%
	61 and above	11	2.2%
Gender	Female	318	63.6%
	Male	182	36.4%
Marital status	Divorced	16	3.2%
	Married	395	79%
	Seperated	4	0.8%
	Single	76	15.2%
	Widowed	9	1.8%
Religion	Christianity	306	61.2%
	Islam	187	37.4%
	None	1	0.2%
	Traditional	6	1.2%
Geopolitical zones	North Central	36	7.2%
	North East	6	1.2%
	North West	11	2.2%
	South East	120	24%
	South South	62	12.4%
	South West	265	53%
Region	Northern Nigeria	53	10.6%
	Southern Nigeria	447	89.4%
Level of education	None	5	1%
	Primary level	50	10%
	Secondary level	171	34.2%
	Tertiary level	274	54.8%
Estimated monthly income	<#50,000	202	40.4%
	#50001-#100,000	145	29%
	#100001-#200,000	78	15.6%
	>#200,000	75	15%
Designation	Both	74	14.8%
	Caregiver/Guardian	74	14.8%
	Parent	352	70.4%
Total number of children	1	131	26.2%
	2	146	29.2%
	3	115	23%
	4	60	12%
	5	30	6%
	> 5	18	3.6%
Ever been diagnosed with COVID	Yes	40	8%
	No	460	92%
Know anyone diagnosed with COVID?	Yes	143	28.6%
	No	357	71.4%
Overall knowledge	Good	265	53%
	Poor	235	47%
Do you think you are at risk of COVID-19 infection?	Yes	169	33.8%
	No	253	50.6%
	Uncertain	78	15.6%
Do you think your child is at risk of COVID-19 infection?	Yes	157	31.4%
	No	257	51.4%
	Uncertain	86	17.2%
Did you previously vaccinate your child from birth	Yes	421	84.2%
	No	79	15.8%
Do you know anyone who has gotten the COVID-19 vaccine?	Yes	269	53.8%
	No	231	46.2%
Overall attitude	Good	266	53.2%
	Poor	234	46.8%
Previous bad experience with vaccination?	Yes	54	10.8%
	No	446	89.2%
Are you willing to accept COVID-19 vaccine for yourself?	Yes	285	57%
	No	121	24.2%
	Uncertain	94	18.8%
Are you willing to accept COVID-19 vaccine for your child?	Yes	242	48.4%
	No	143	28.6%
	Uncertain	115	23%

Among 500 respondents, 242 (48.4%) were willing to vaccinate their child and 285 (57%) respondents were willing to vaccinate themselves against COVID-19 disease while 23 and 18.8% were unsure of vaccinating their child and themselves against the disease (Table 1). Most parents/caregivers felt that healthcare workers were the most credible source of information regarding COVID-19, followed by mass media (Television/Radio). Social media was regarded as the least credible source of information (Table 2). Approximately half of the surveyed parents/caregivers (58.2%) knew that COVID-19 is preventable with vaccines; however, 73% believed that the COVID-19 vaccine can be used to protect oneself against severe disease. Only 56.8% of surveyed respondents believed that receiving the COVID-19 vaccine can be used to protect other family members (Table 3).

**Table 2:** Information sources about COVID-19 and COVID-19 vaccine.

Source of information	Do not use this	Not credible at all	Not too credible	Very credible
Social media (Facebook/Instagram/ WhatsApp)	74 (14.8%)	54 (10.8%)	184 (36.8%)	188 (37.6%)
Television/Radio	16 (3.2%)	26 (5.2%)	111 (22.2%)	347 (69.4%)
Newspapers	71 (14.2%)	39 (7.8%)	124 (24.8%)	266 (53.2%)
Family/Friends/Colleagues	25 (5.0%)	60 (12.0%)	221 (44.2%)	194 (38.8%)
Teachers	66 (13.2%)	42 (8.4%)	194 (38.8%)	198 (39.6%)
Religious leaders	41 (8.2%)	60 (12.0%)	184 (36.8%)	215 (43.0%)
Political leaders	70 (14.0%)	143 (28.6%)	168 (33.6%)	119 (23.8%)
Healthcare workers	33 (6.6%)	26 (5.2%)	77 (15.4%)	364 (72.8%)

**Table 3:** Participant’s knowledge about COVID-19 and COVID-19 vaccine.

Variable		Frequency	Percentage
COVID-19 is a Respiratory Infection?	Yes	358	71.6%
	No	30	6%
	I do not know	112	22.4%
COVID-19 can be transmitted via respiratory droplets and contact with infected persons	Yes	392	78.4%
	No	24	4.8%
	I do not know	84	16.8%
Symptoms of COVID-19 include fever, cough, chest pain and fatigue?	Yes	381	76.2%
	No	28	5.6%
	I do not know	91	18.2%
All patients have symptoms	Yes	137	27.4%
	No	232	46.4%
	I do not know	131	26.2%
COVID-19 develops within 2-14 days	Yes	263	52.6%
	No	62	12.4%
	I do not know	175	35%
Children cannot get COVID-19	Yes	85	17%
	No	279	55.8%
	I do not know	136	27.2%
COVID-19 is preventable with the use of vaccines	Yes	291	58.2%
	No	65	13%
	I do not know	144	28.8%
COVID-19 vaccine can be used to protect oneself	Yes	365	73%
	No	37	7.4%
	I do not know	98	19.6%
COVID-19 vaccine is used by government to steal money	Yes	56	11.2%
	No	260	52%
	I do not know	184	36.8%
COVID-19 vaccine can be used to protect family from the disease	Yes	284	56.8%
	No	59	11.8%
	I do not know	157	31.4%

Less than half of the respondents agreed that the COVID-19 vaccine is safe and effective; however, when queried on vaccines in general, majority of them (77.6%) agreed that vaccines are generally important in children. Eighty-two respondents (16.4%) reported that they do not believe in vaccines while 231 respondents (46.2%) reported that they would not vaccinate their child as they are worried about the side effects of the vaccine (Table 4). It is noteworthy that having a good attitude toward COVID-19 vaccination was significantly associated with willingness to vaccinate oneself against the disease (*p* < 0.001). Likewise, respondents who had a good attitude toward COVID-19 vaccination were also found to be six times more likely to be willing to vaccinate their child compared to those who had a poor attitude toward COVID-19 vaccination, and this relationship was statistically significant.

**Table 4:** Participants attitude toward COVID-19 and COVID-19 vaccine.

Variable		Frequency	Percentage
COVID-19 vaccine is safe and effective?	Agree/Strongly agree	220	44%
	Disagree/Strongly Disagree	61	12.2%
	Neutral	219	43.8%
Are you scared of contracting COVID-19 disease?	Agree	147	29.4%
	Disagree	226	45.2%
	Neutral/Uncertain	127	25.4%
Vaccines are important in children	Agree	388	77.6%
	Disagree	34	6.8%
	Neutral/Uncertain	78	15.6%
I trust vaccines and health workers	Agree	337	67.4%
	Disagree	46	9.2%
	Neutral/Uncertain	117	23.4%
I do not believe in vaccines generally	Agree	82	16.4%
	Disagree	323	64.6%
	Neutral/Uncertain	95	19%
I do not want my child to get the vaccine	Agree	366	73.2%
	Disagree	76	15.2%
	Neutral/Uncertain	58	11.6%
I do not trust the vaccine	Agree	119	23.8%
	Disagree	217	43.4%
	Neutral/Uncertain	164	32.8%
The vaccine is from the devil	Agree	33	6.6%
	Disagree	347	69.4%
	Neutral/Uncertain	120	24%
The vaccine is a micro-chip to control the world	Agree	49	9.8%
	Disagree	313	62.6%
	Neutral/Uncertain	138	27.6%
I will vaccinate my child to protect him/her	Agree	259	51.8%
	Disagree	95	19%
	Neutral/Uncertain	146	29.2%
I will vaccinate my child to protect other family members	Agree	271	54.2%
	Disagree	97	19.4%
	Neutral/Uncertain	132	26.4%
I will vaccinate my child because of school	Agree	263	52.6%
	Disagree	101	20.2%
	Neutral/Uncertain	136	27.2%
I will not vaccinate my child as I am worried about the side effects of vaccine	Agree	231	46.2%
	Disagree	96	19.2%
	Neutral/Uncertain	173	34.6%
I believe the vaccines are fake	Agree	59	11.8%
	Disagree	233	46.6%
	Neutral/Uncertain	208	41.6%

Willingness to vaccinate self was strongly associated with willingness to vaccinate their child (*p* < 0.001). Only 0.5% of those who did not want to vaccinate themselves were willing to vaccinate their children while 84.6% of those willing to vaccinate themselves were willing to vaccinate their children. Good knowledge and attitude were also strongly associated with willingness to vaccinate children (*p* < 0.001 and *p* < 0.001 respectively) (Table 5).

**Table 5:** Factors associated with willingness to vaccinate child against COVID-19.

Variables		Willing to vaccinate child (*n* = 242)	Unwilling to vaccinate child (*n* = 262)	Total (*n* = 500)	*X* ^2^	*p* value
Age	>40 years	107 (57.2%)	80 (42.8%)	187 (100%)	9.303	0.002
	<=40 years	135 (43.1%)	178 (56.9%)	313 (100%)		
Gender	Male	112 (61.5%)	70 (38.5%)	182 (100%)	19.779	<0.001
	Female	130 (40.9%)	188 (59.1%)	318 (100%)		
Marital status	Married	192 (48.6%)	203 (51.4%)	395 (100%)	0.032	0.857
	Others	50 (47.6%)	55 (52.4%)	105 (100%)		
Religion	Christian	142 (46.4%)	164 (53.6%)	306 (100%)	1.257	0.262
	Others	100 (51.5%)	94 (48.5%)	194 (100%)		
Geopolitical zone	North Central	9 (25.0%)	27 (75.0%)	36 (100%)	14.655	0.0093*
	North East	4 (66.7%)	2 (33.3%)	6 (100%)		
	North West	5 (45.5%)	6 (54.5%)	11 (100%)		
	South East	50 (41.7%)	70 (58.3%)	120 (100%)		
	South South	30 (48.4%)	32 (51.6%)	62 (100%)		
	South West	144 (54.3%)	121 (45.7%)	265 (100%)		
Region	Southern Nigeria	224 (50.1%)	223 (49.9%)	447 (100%)	4.948	0.026
	Northern Nigeria	18 (34%)	35 (66%)	53 (100%)		
Highest level of education	Tertiary Level	141 (51.5%)	133 (48.5%)	274 (100%)	2.273	0.132
	Others	101 (44.7%)	125 (55.3%)	226 (100%)		
Monthly income	>$100	148 (49.7%)	150 (50.3%)	298 (100%)	0.472	0.492
	<=$100	94 (46.5%)	108 (53.5%)	202 (100%)		
Number of children	>2	108 (48.9%)	113 (51.1%)	221 (100%)	0.035	0.852
	<=2	134 (48%)	145 (52%)	279 (100%)		
Previous COVID-19 infection	Yes	21 (52.5%)	19 (47.5%)	40 (100%)	0.293	0.589
	No	221 (48%)	239 (52%)	460 (100%)		
Know anyone diagnosed with COVID-19	Yes	81 (56.6%)	62 (43.4%)	143 (100%)	5.449	0.020
	No	161 (45.1%)	196 (54.9%)	357 (100%)		
Overall, knowledge	Good	168 (61.5%)	105 (38.5%)	273 (100%)	41.562	<0.001
	Poor	74 (32.6%)	153 (67.4%)	227 (100%)		
Feeling at risk of COVID-19	Yes	117 (69.2%)	52 (30.8%)	169 (100%)	44.355	<0.001
	No	125 (37.8%)	206 (62.2%)	331 (100%)		
Feeling child is at risk of COVID-19	Yes	109 (69.4%)	48 (30.6%)	157 (100%)	40.516	<0.001
	No	133 (38.8%)	210 (61.2%)	343 (100%)		
Previously vaccinated child	Yes	208 (49.4%)	213 (50.6%)	421 (100%)	1.08	0.299
	No	34 (43%)	45 (57%)	79 (100%)		
Know COVID-19 vaccinated person	Yes	156 (58%)	113 (42%)	269 (100%)	21.453	<0.001
	No	86 (37.2%)	145 (62.8%)	231 (100%)		
Willingness to vaccinate self	Yes	241 (84.6%)	44 (15.4%)	285 (100%)	347.034	<0.001*
	No	1 (0.5%)	214 (99.5%)	215 (100%)		
Attitude	Good	202 (76%)	64 (24%)	266 (100%)	172.6	<0.001
	Poor	40 (17.1%)	194 (82.9%)	234 (100%)		
Previous bad experience with vaccination	No	167 (49.7%)	169 (50.3%)	336 (100%)	0.829	0.363
	Yes	73 (45.3%)	88 (54.7%)	161 (100%)		

Willingness to vaccinate self was strongly associated with willingness to vaccinate their child. Only 0.5% of those who did not want to vaccinate themselves were willing to vaccinate their children while 84.6% of those willing to vaccinate themselves were willing to vaccinate their children. Good knowledge and attitude were also strongly associated with willingness to vaccinate children (Table 5).

Univariate analysis also showed that age greater than 40 years, male gender, knowledge of a person previously diagnosed with COVID-19, being from Southern Nigeria, having good knowledge of COVID-19 disease and vaccine, having good attitude toward COVID-19 vaccination, presence of perceived risk of COVID-19 infection for themselves and risk of infection for their child, knowledge of someone who had gotten vaccinated and willingness to vaccinate self were significantly associated with higher odds of parental vaccine acceptability. After adjusting for covariates, willingness to vaccinate self, good attitude and age greater than 40 years were still associated with higher odds of vaccine acceptance. Compared to those unwilling to vaccinate themselves, those who were willing to receive the vaccine were a thousand times more likely to vaccinate their child [AOR: 1016.81; 95% CI = (128.51–8045.60)]. People aged greater than 40 years and those with good attitude were twice more likely and six times more likely to accept the vaccine than those aged younger than 40 years [AOR: 2.56; 95% CI = (1.14–5.76)], and those with bad attitude, respectively [AOR: 6.21; 95% CI = (2.83–13.64)] (Table 6).

**Table 6:** Bivariate logistic analysis of factors associated with willingness to vaccinate child.

Characteristics	Adjusted odds ratio (95% CI)	*p* value
Age greater than 40 years	2.56 (1.14 to 5.76)	**0.023**
Male gender	1.98 (0.94 to 4.2)	0.073
Residing in Southern Nigeria	1.47 (0.45 to 4.8)	0.520
Knowing an infected person	0.45 (0.18 to 1.08)	0.075
Good knowledge	0.65 (0.25 to 1.67)	0.371
Feeling at risk of COVID-19 Infection	1.96 (0.72 to 5.33)	0.185
Feeling child is at risk of COVID-19 Infection	1.23 (0.45 to 3.4)	0.686
Knowing a vaccinated person	0.69 (0.27 to 1.81)	0.455
Willingness to vaccinate self	1016.81 (128.51 to 8045.6)	**<0.001**
Good attitude	6.21 (2.83 to 13.64)	**<0.001**

*Bold here refers to those values that were statistically significant.

## Discussion

Our study showed that less than half of Nigerian parents are willing to vaccinate their children against COVID-19. This is much lower than what obtains in high income countries (21, 22). Bianco et al. (21) found that 82% of parents surveyed in Italy were willing to vaccinate their children against COVID-19 while Goldman et al. (22) found that 61.1% of parents surveyed in Canada were willing to vaccinate their child. We found that willingness to vaccinate self was a strong predictor of willingness to vaccinate their children, which is similar to previous findings (1, 23–25). However, more than one-tenth of respondents who were willing to vaccinate themselves were still not willing to vaccinate their children, alluding to the need for intensified parental education about COVID-19 vaccine’s safety and efficacy in children to ensure uptake when it is rolled out. We also found that having good attitude toward COVID-19 vaccination and good knowledge of COVID-19 disease was associated with willingness to vaccinate their child. This is in line with findings from a similar study by Hunyh et al. that examined vaccine hesitancy amongst the general population and parents (23).

In our study, older parents/caregivers aged greater than 40 years were more likely to vaccinate their child against COVID-19, similar to previous findings (2, 26). In a scoping review that summarized the evidence on vaccine hesitancy in Africa, it was reported that being a male was associated with positive attitude toward the vaccine (27). Our study also showed that men were more likely to vaccinate their child, similar to previous studies that have reported women to be less likely to be willing to vaccinate their child (1, 28). Gender disparities across various sectors of the economy in Nigeria, characterized by lower rates of female labor force participation and inadequate investment in women’s human capital, may account for the greater likelihood of men vaccinating their children compared to women (29). This disparity translates to men having enhanced access to healthcare information, and as such, may be a key factor responsible for their increased inclination toward vaccinating their children. In order to convince men who are disinclined to vaccinate their children, healthcare providers should be encouraged to employ diverse strategies such as targeted educational interventions via social media and inclusive involvement of fathers in the vaccination decision-making process for children from infancy. We believe this may be due to fear of adverse side effect of the virus amongst women. Further research to investigate specific cause of this gender difference should be done and public health professionals should develop gender-specific messages to promote vaccine acceptance amongst women.

Interestingly, the present study did not find a significant relationship between level of education, marital status, and willingness to vaccinate children. This contrasts with previous findings that identified that parents with secondary or lower education and those who were single were more willing to vaccinate their child against COVID-19 (21, 30). Padhi et al. (25) in their study reported conflicting findings that people with higher levels of education were more willing to vaccinate their child (25). However, this was not observed in our study; this may be because our surveyed population mainly consisted of young parents age less than 40 years. This inconsistency has been previously reported when evaluating the educational level of parents and intention to vaccinate; hence it is possible that education may not always be a key determinant of willingness to accept vaccine (19).

In addition to the previously discussed sociodemographic factors related to parental willingness to vaccinate their child, we also investigated the role of the perceived risk of COVID-19 infection. Past research has shown that people who perceived they or their child were at risk of getting infected with COVID-19 were more likely to be willing to vaccinate their child (31, 32). In this present study, we also found that parents who perceived themselves or their child could get infected with the SARS-CoV-2 virus were more willing to accept the vaccine. Majority of our respondents found healthcare workers to be the most credible source of information regarding COVID-19. Public health campaigns aiming to improve willingness need to find ways to tailor their message using healthcare workers for community outreaches and campaigns on mass media platforms. Future research should investigate the impact of information sources on parental vaccination intention.

### Strength and limitations

The strength of this study is that it was a nationwide survey of parents and caregivers with children in Nigeria. The COVID-19 vaccine was recently approved for use among 16- and 17-year-old Nigerian children for educational and travel purposes, and it is likely that in the light of the recent fear of the fifth wave of the virus, the vaccine may soon be expanded to include other children older than 12 years. Hence, the findings of our survey will be useful to guiding public health professionals and other stakeholders on targeted campaigns to promote vaccine uptake amongst Nigerian children.

However, our findings should be interpreted in the light of certain limitations. Firstly, it was an internet-based survey and, as such is subject to selection bias. People who may have participated are internet-savvy and are likely to be more educated than the average Nigerian and as such, the generalizability of our findings is limited. Further research using the identified factors related to vaccine acceptability should consider targeting people with no access to the internet. Secondly, this was a cross-sectional survey, and we cannot infer causality for the reported associations. Third, according to the data collected, respondents that took this survey were mostly from southern Nigeria. Northern Nigeria was the least represented and seeing as vaccine hesitancy is rifer in that region, it is possible that our findings may not be applicable to parents and caregivers in that part of Nigeria. Further research to investigate their willingness to accept COVID-19 vaccine for their children and associated factors is recommended. Fourth, our study did not explore the impact of co-morbidities on parental willingness. Studies investigating the role of co-morbidities as a determinant in vaccine acceptance have reported conflicting findings. While some have shown that parents were less willing to vaccinate a child with co-morbidities (23), others have shown parent’s being more willing to vaccinate a child with co-morbidities (33). These studies have mostly been done in high income countries and as such, there is a need to replicate it in the African population. Lastly, our study did not investigate the impact of the various types of information sources on parental vaccination intention and as such, we cannot determine whether it influences parental decision.

## Conclusion

The present study investigated the knowledge, attitude, and willingness of Nigerian parents to vaccinate their children against COVID-19 disease. It was shown that less than half of parents were willing to vaccinate their child. We found that willingness to vaccinate self, being aged greater than 40 years and a good attitude toward COVID-19 vaccine were significant predictors of vaccine acceptability. In the context of expanding COVID-19 vaccine access to children in the future, the results point to the importance of considering these factors in the design of evidence-based vaccine promotion campaigns targeted toward improving uptake.

## Data availability statement

The raw data supporting the conclusions of this article will be made available by the authors, without undue reservation.

## Author contributions

AA, CA, AI, TD, TK, BR, MA, OO, YS, and AR: idea, design, and editing. AA, CA, AI, TD, and AR: prepared the questionnaires. AA, CA, and AI: analyzes. All authors collected the data, wrote the draft, and approved the final draft.

## Funding

This study was funded by the Neo Child Initiative, Lagos, Nigeria.

## Conflict of interest

The authors declare that the research was conducted in the absence of any commercial or financial relationships that could be construed as a potential conflict of interest.

## Publisher’s note

All claims expressed in this article are solely those of the authors and do not necessarily represent those of their affiliated organizations, or those of the publisher, the editors and the reviewers. Any product that may be evaluated in this article, or claim that may be made by its manufacturer, is not guaranteed or endorsed by the publisher.
